# The development and validation of a dashboard prototype for real-time suicide mortality data

**DOI:** 10.3389/fdgth.2022.909294

**Published:** 2022-08-20

**Authors:** R. Benson, C. Brunsdon, J. Rigby, P. Corcoran, M. Ryan, E. Cassidy, P. Dodd, D. Hennebry, E. Arensman

**Affiliations:** ^1^ School of Public Health, College of Medicine and Health, University College Cork, Cork, Ireland; ^2^ National Suicide Research Foundation, WHO Collaborating Centre for Surveillance and Research in Suicide Prevention, Cork, Ireland; ^3^ National Centre for Geocomputation, National University of Ireland Maynooth, Maynooth, Ireland; ^4^ Cork Kerry Community Health Services, Health Service Executive, Cork, Ireland; ^5^ Department of Psychiatry and Neurobehavioural Science, University College Cork, Cork, Ireland; ^6^ National Office for Suicide Prevention, Health Service Executive, Dublin, Ireland

**Keywords:** real-time, suicide, surveillance, dashboard, data visualisation, cluster detection

## Abstract

**Introduction/Aim:**

Data visualisation is key to informing data-driven decision-making, yet this is an underexplored area of suicide surveillance. By way of enhancing a real-time suicide surveillance system model, an interactive dashboard prototype has been developed to facilitate emerging cluster detection, risk profiling and trend observation, as well as to establish a formal data sharing connection with key stakeholders *via* an intuitive interface.

**Materials and Methods:**

Individual-level demographic and circumstantial data on cases of confirmed suicide and open verdicts meeting the criteria for suicide in County Cork 2008–2017 were analysed to validate the model. The retrospective and prospective space-time scan statistics based on a discrete Poisson model were employed *via* the R software environment using the “*rsatscan*” and “*shiny”* packages to conduct the space-time cluster analysis and deliver the mapping and graphic components encompassing the dashboard interface.

**Results:**

Using the best-fit parameters, the retrospective scan statistic returned several emerging non-significant clusters detected during the 10-year period, while the prospective approach demonstrated the predictive ability of the model. The outputs of the investigations are visually displayed using a geographical map of the identified clusters and a timeline of cluster occurrence.

**Discussion:**

The challenges of designing and implementing visualizations for suspected suicide data are presented through a discussion of the development of the dashboard prototype and the potential it holds for supporting real-time decision-making.

**Conclusions:**

The results demonstrate that integration of a cluster detection approach involving geo-visualisation techniques, space-time scan statistics and predictive modelling would facilitate prospective early detection of emerging clusters, at-risk populations, and locations of concern. The prototype demonstrates real-world applicability as a proactive monitoring tool for timely action in suicide prevention by facilitating informed planning and preparedness to respond to emerging suicide clusters and other concerning trends.

## Introduction

Approximately 703,000 lose their life to suicide each year worldwide (1). Reducing the global incidence of suicide is an international priority (2–4). The Comprehensive Mental Health Action Plan 2013–2030 of the World Health Organization (WHO) and The United Nations Sustainable Development Goal 3 includes the specific target to reduce premature mortality by non-communicable diseases by one third, basing the rate of suicide mortality per 100,000 of the population as an indicator (2, 3). To monitor progress towards this target, it is pertinent to obtain and collate suicide mortality data in a timely manner to inform the implementation, adaption, and evaluation of suicide prevention strategies to ensure their efficiency and efficacy in achieving the important objective of reducing deaths by suicide (5). To optimize utility, suicide mortality data should be disaggregated at least by gender, age, and method of death (6). Accessibility to current suicide mortality data has additional benefits including early identification of emerging suicide contagion and clusters, a timely response to people affected by suicide, and verification of anecdotal evidence and public statements that are disseminated *via* media outlets (5, 7). Furthermore, real-time suicide surveillance facilitates timely action in response to emerging situations that may impact rates of suicide (5). The demand for such data has grown significantly since the onset of the Coronavirus (COVID-19) pandemic, during which many nations have depended on drawing inferences on the impact of the pandemic and relevant societal restrictions on suicide rates based on dated statistics, due to a dearth (8). The WHO endorses the establishment of long-term, continuous, and sustainable surveillance of suicide and self-harm either prior to or upon commencing implementation of a national suicide prevention strategy, suggesting that countries wherein resources are limited, piloting may be conducted at regional level with the intent to scale up to continuous data collection for surveillance purposes (6).

Monitoring suicide on a national scale presents many challenges including geographical inconsistencies in investigative approaches and timeliness of mortality data made available by official statistical agencies (9). In Ireland, the process of verification, registration and classification of external causes of death, including suicides, usually involves several months and in some cases up to two years due to the requirement of a Coroner's inquest and the involvement of An Garda Síochana, pathologists, and Vital Statistics Registrars, which can also result in late registered suicide deaths (10). In response to a specific objective of Ireland's national suicide prevention strategy Connecting for Life 2015–2024 (11), to improve access to timely and high-quality data on suicide and self-harm, a real-time suicide surveillance system was established in the Southwest region of Ireland. The Suicide and Self-Harm Observatory (SSHO), a unique data system, collates provisional data on deaths by suspected suicide on a continuous basis in counties Cork and Kerry (12). De-identifiable, disaggregated data based on demographic details of the deceased individual and circumstantial information surrounding the death are collated for the purpose of trend analysis and aberration detection, including the emergence of suicide contagion and clusters. To support and enhance the objectives of the SSHO, an interactive dashboard has been developed to visually display the data and provide an analytic tool that has access restricted to key stakeholders within suicide prevention.

Dashboards have been used extensively within public health surveillance and serve as data visualisation and dissemination tools that facilitate exploratory data analysis and data-driven decision making (13). The tools support better interaction with data by compiling, aggregating, and filtering relevant information in an efficient manner, making it more readily consumable (14). Utilising real-time suspected suicide data can only enhance the functionality of a dashboard by disseminating suicide mortality data at the earliest opportunity from which trends, at-risk populations and geographic variability may be examined to inform tailored prevention efforts (8).

Considerable advances have been made in investigations of the presence of space-time clusters of non-communicable diseases within a population (15). Both statistical and mapping techniques have been extensively applied to retrospective suicide mortality data (16–18). Prospective spatial scanning methodology has been used in geographical disease surveillance to detect emerging clusters (19), although it has not yet been applied to suicide mortality data. The prospective application of such techniques to real-time suicide mortality data, using parameters applicable to non-communicable diseases, would support the identification of emerging or active clusters, thereby offering the opportunity for early intervention, mitigation of further cases and facilitation of targeted response efforts in affected communities. The incorporation of Geographical Information Systems (GIS) provides the means to perform such surveillance functions by geocoding cases for rate calculations and modelling space-time patterns (20). Furthermore, opensource GIS software offers the opportunity to develop a low-cost, sustainable surveillance system, ensuring that developing countries with restrained resources can also implement a sustainable surveillance model (21, 22). To date, there is no available scientific literature to suggest the implementation of GIS in real-time suicide surveillance, demonstrating innovation in such an approach.

Employing the space-time scan statistic, this study aims to demonstrate a workable dashboard prototype by determining the performance of the modelling feature based on retrospective suicide mortality data collated by the Suicide Support and Information System [SSIS; (23–26)] in County Cork, Ireland, over a ten-year period. To our knowledge, this is the first study to investigate the presence of suicide clusters in a population on an ongoing basis using the prospective space-time scan statistic. In validating the dashboard prototype, we aim to establish an evidence-based model that provides predictive analytics in a user-friendly web-based interface to inform suicide prevention interventions.

## Methods and materials

### Data

#### Suicide support and information system

A dataset including a total of 388 cases of confirmed suicide and open verdicts that meet the criteria for death by suicide (27) which occurred in County Cork between 1 January 2008 and 31 December 2017 were collated by means of psychological autopsy of coronial records by the SSIS (23–26). The geographical boundary on which the data have been collated for this study is that imposed by Cork County and City Councils up until 2018 at which time the county and city boundaries were revised. Residential address geographical coordinates and data on the date of death of the decedents were included for all cases, as well as additional data including the date of birth, gender, method of death, presence of note, history of self-harm, history of psychiatric inpatient care and employment background. To generate the required geographical coordinates data for spatial analysis, the latitude and longitude of the residential address of each case were extracted, using the *taRifx.geo* R package, and Microsoft Bing's geocoding facility.

#### Census data

Small-area level population density data was sourced from the Central Statistics Office (28), facilitating analysis at the smallest unit possible. To generate the required population file, the data reflects population counts for each small-area level in County Cork from the most recently available Census data in 2016.

### Analysis

SaTScan v10.0.2, a free software that uses spatial, temporal or spatio-temporal scan statistics to detect disease clusters and determine their statistical significance was employed in both prospective and retrospective mode (29). SaTScan was executed using an R software package known as “*rsatscan”* v0.3.9200, which acts as a wrapper for SaTScan ensuring easy automation, integration, and less computational intensity (30).

#### Retrospective spatio-temporal scan statistic mode

To detect clusters retrospectively, SaTScan was run in batch mode *via* the R rsatscan package using all 10-years of available surveillance data for the period 2008–2017. The spatio-temporal scan statistic, employing a moving circular geographical-based scan window and a time-based height dimension of continuously varying radius, was executed in batch mode. The parameters were repeatedly manipulated to determine the best-fit model of a 30-day temporal window, based on a one-day time aggregation. Standard Monte Carlo simulations (999) were employed to calculate the statistical significance of detected clusters with a *P*-value <0.05 deemed statistically significant. SaTScan computes the relative risk (RR) for each detected cluster.

#### Prospective spatio-temporal scan statistic mode

To simulate real-time cluster detection, we employed the prospective spatio-temporal analysis mode. This approach involves repeated analysis on a daily, weekly, monthly, or yearly basis for early detection of emerging clusters (31). The scan statistic was executed as if it had been performed throughout the period of 2008–2017. Other than the use of the prospective space-time analysis mode, the parameters remained the same from the retrospective analysis mode.

### Output

**Figure 1:**
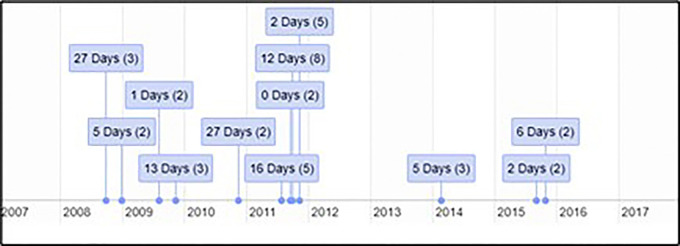
Timeline of suicide clusters occurring in County Cork during the period 2008–2017.

R version 4.0.4, a free and open-source statistical software, and the RStudio version 1.4.1106, an IDE interface provide an environment and programming language for data manipulation, calculation, and graphical display. *Shiny*, another R package, facilitates development of both static and interactive tools. The output of the rsatscan cluster analysis was plotted by means of an Environmental Systems Research Institute (ESRI) shapefile, produced to display the detected clusters in a map format (Figure 1). To illustrate the utility of a data visualisation tool, a timeline feature was designed through which the user can select a cluster on the timeline to display the cluster on the map. It can display outputs and build reactive plots and tables, offering timeseries and mapping features.

## Results

**Figure 2:**
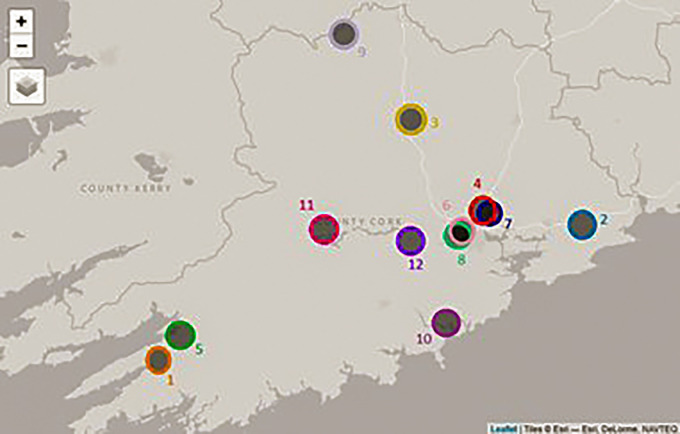
Map of suicide clusters occurring in County Cork during the period 2008–2017.

**Figure 3:**
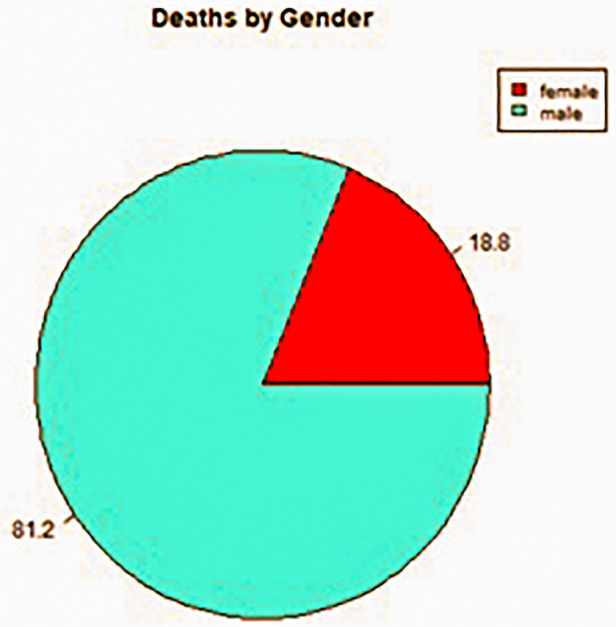
Gender breakdown of deaths by suicide during the period 2008–2017 in County Cork.

**Figure 4:**
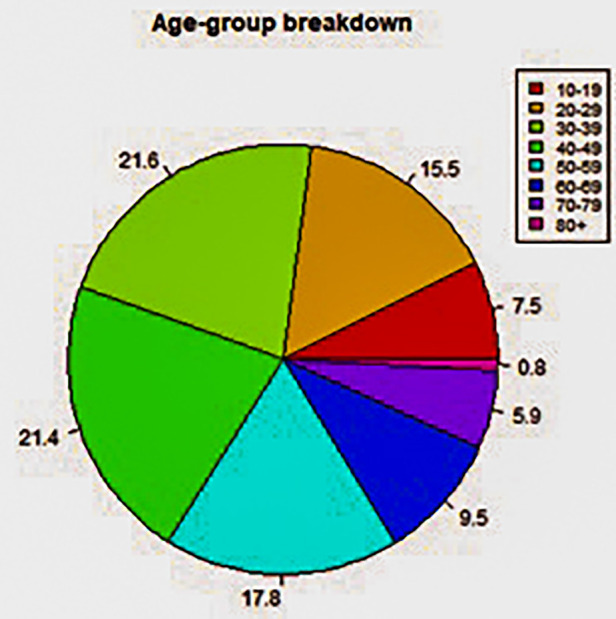
Age-group breakdown of deaths by suicide during the period 2008–2017 in County Cork.

**Figure 5:**
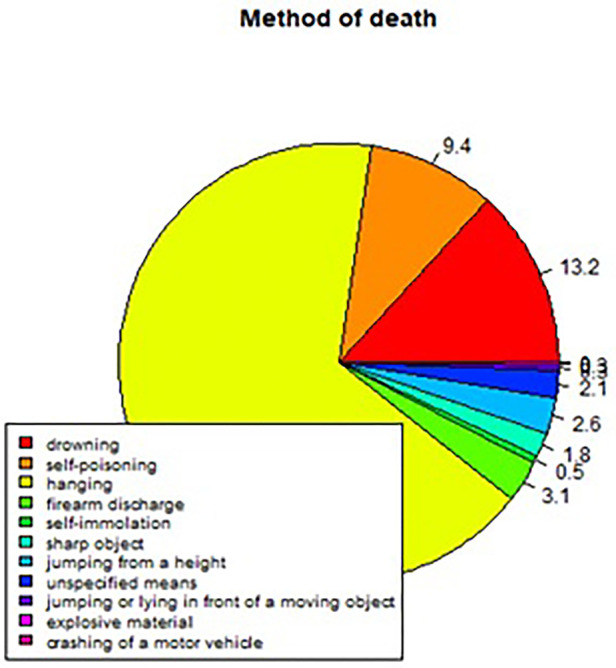
Percentage breakdown of the method of deaths by suicide during the period 2008–2017 in County Cork.

The retrospective space-time analysis mode detected 12 clusters, none of which were of statistical significance (*P* < 0.05; Table 1). The mean number of cases in a detected cluster was 2, which was the minimum case count in the parameters applied in the scan statistic investigation. No clusters were detected by the prospective space-time analysis mode. Analytical outputs were produced by R, including a timeline and static map with plotted clusters detected by SaTScan, as well as disaggregated gender and age breakdowns of deaths by suicide in County Cork during the period 2008–2017 (Figures 1–5).

**Table 1:** Clusters detected during the period 2008–2017 in County Cork.

Cluster	Start date	End date	Duration of cluster (days)	Expected number of cases	Observed number of cases	*P-*value	Test statistic
1	2011/09/15	2011/09/15	0	0.010	2	0.111	8.551
2	2015/10/27	2015/11/2	6	0.010	2	0.498	8.551
3	2009/08/04	2009/08/05	1	0.015	2	0.852	7.745
4	2008/12/27	2009/01/01	5	0.015	2	0.852	7.745
5	2011/09/28	2011/10/10	12	0.103	3	0.854	7.226
6	2010/11/14	2010/12/11	27	0.021	2	0.934	7.175
7	2009/11/10	2009/11/23	12	0.023	2	0.956	6.942
8	2014/02/20	2014/02/25	5	0.023	2	0.956	6.942
9	2015/09/03	2015/09/05	2	0.026	2	0.978	6.733
10	2011/11/09	2011/11/11	2	0.026	2	0.978	6.733
11	2011/07/26	2011/08/11	16	0.039	2	0.998	5.936
12	2008/09/25	2008/10/22	27	0.046	2	0.998	5.579

## Discussion

The current study sought to demonstrate a workable prototype of a real-time public health surveillance model to detect suicide clusters or emerging clusters, employing space-time scanning techniques and graphic display means to disseminate data in a visually supportive manner to inform decision-making in suicide prevention. The findings prove that the retrospective space-time scan statistic has feasibility to detect emerging clusters using geocoded data. The results also prove the capability of R software and specifically the rsatscan and shiny packages in running the analysis and utilising the analytic output to produce data visualisations such as static and interactive cluster maps and timelines, as well as percentage breakdowns of gender, age, and methods of death by suicide in pie chart format, demonstrating its compatibility as a dashboard platform for the SSHO.

Irrespective of the income level of a country, a common challenge in conducting surveillance, particularly in real-time, is the availability of accurate data (30). This is particularly relevant in the context of spatio-temporal analyses which incorporate data on the population at risk as a divisor in risk estimates (31). In small-area level investigations of mortality, resident population counts are often sourced from national census data, which creates issues in intercensal estimate calculations, thereby inhibiting accurate data representations of the population. Furthermore, limitations in access to current, non-aggregated suicide mortality data result in outdated surveillance and cluster investigations that inform less effective response measures due to the retrospective nature of the data analysed. The SSHO overcomes this limitation by collating routine, individual-level data from the primary source, using a strict criterion to ensure surveillance is based on complete and current data. As such, the SSHO serves as a reliable data source to verify unsubstantiated reports and dispel misinformation circulated by traditional and social media relating to suspected suicide rates and emerging suicide clusters in the region. Data displayed by the SSHO dashboard will represent a current profile of individuals who have died by suspected suicide in the region. In essence, the dashboard can provide graphic support to timely, data-driven policy development, implementation, and evaluation, thereby utilising existing resources effectively and allocating additional resources in identified priority areas.

The type of software employed in a surveillance system is integral to its functionality. While GIS can plot the spatial variation among cases, they are not designed to manage temporal data. Thus, statistical analysis of spatio-temporal data is essential to identify aberrations in underlying disease occurrence (32). Comparisons of space-time surveillance approaches have found SaTScan to be the least complex, most user-friendly, and best equipped package for automated surveillance purposes (32, 33). Data pre-processing, i.e., formatting data to fit the requirements of surveillance software package analysis can be time consuming. A key advantage of SaTScan is the option to aggregate data temporally, thereby reducing the data restructuring required when applying different parameters and accepting data input at its finest temporal resolution. This unique feature is particularly useful for prospective analysis which involves multiple time periodic analyses. In addition, SaTScan conducts cluster detection analysis at a higher speed than other surveillance software programs. One limitation of the software is the lack of inbuilt data exploration function and data outputs are limited to database and text files (32). This constraint has been overcome in the present research by employing SaTScan *via* the R rsatscan package to conduct the cluster detection investigation and programming the visual display of the analytical output using the shiny package. The features of R shiny may be extended to deploy the surveillance system output to the stakeholders for which the dashboard has been built to serve. Utilising free and opensource software, as explored in the current research, provides a suitable and sustainable platform for a surveillance system. Sharing research code is standard practice in many fields of research and has many benefits, including promoting standardisation, and avoiding duplication and waste of resources (33). This approach ensures that the system framework is widely accessible to low- and middle-income countries and regions that may be lacking budgetary support to meet the resource needs for a well-functioning real-time suicide surveillance system (34).

Real-world investigations of space-time signals can be cumbersome and inefficient (34). A major challenge involved in analysing large and complex datasets that include space and time data items is the computational burden, especially in the amount of small-area level units examined which can vary depending on the spatial and temporal resolutions selected, even more so when more than one outcome is analysed concurrently (31). Combining the standalone SaTScan software with R *via* rsatscan increases the processing speed of analysis (35). This also ensures the data is automatically updated on a defined periodical basis, avoiding delay in data input to the system and immediate reflection on the dashboard, thereby accelerating data collation to overcome time lags associated with official national statistical reports.

An understanding of R programming language is required to run an analysis of such complexity. However, development and incorporation of R code to conduct and display clusters means that the computational component may be readily implemented by the surveillance system developers and easily disseminated to and used by other researchers within the field, promoting standardisation for comparative analysis of data from other regions or countries. This is particularly facilitated by the use of the shiny package, which can create interactive interfaces that allow researchers or others involved in surveillance to query the data interactively. Sharing research code is standard practice in many fields of research and has many benefits, including promoting standardisation, and avoiding duplication and waste of resources (36). Since the data collated and presented by the dashboard is based on highly sensitive, provisional data based on cases of suspected suicide that have yet to undergo coronial inquest, data protection, confidentiality and privacy issues must be considered. Encryption, user licence and authorisation requirements would ensure content is only available to stakeholders and research personnel with approved access rights on a need-to-know basis, while also preventing download of raw data underpinning the dashboard. One possibility may be to make analysis tools *via* web sites that are only available internally, and to require secure access *via* a password, or other ID system such as fingerprint or facial recognition.

Data mining and machine learning approaches to geographical data are useful; however, a gap exists between the analytics and the interpretation of results, particularly in anomaly detection (37). The data dashboard provides a functional solution to this issue by presenting analytical outputs through data visualisations including pie charts, time series graphs and maps, accompanied by drill down features, intended to help users comprehend the relationship between the spatial and temporal factors of an event by means of an intuitive interface (31). The current study demonstrates that data visualisations serve the purpose of providing more readily consumable analytical outputs, with the potential to positively impact decision-making in suicide prevention.

The incompatibility of the prospective space-time permutation scan statistic employed in the detection of clusters in this present research was anticipated given that the primary data on which the analyses was simulated on is retrospectively dated, with the most recent case occurring five years prior to the present-day application of the prospective analytic approach. Prospective scan statistic focuses on detecting clusters that emerge or occur at any point during the study period, but remain active on the final day of analysis, thus the prospective analytical approach is conducive and confined to real-time analysis (19). In comparison, the retrospective approach is based on disease mapping of a predefined geographical area over a fixed period (26). This suggests that the prospective spatio-temporal approach would be compatible as a statistical tool for real-time surveillance of suspected suicide in the SSHO and should undergo piloting to determine its efficacy in regular time periodic surveillance of suspected suicide and detection of active or emerging clusters in the South-West region of Ireland.

### Strengths and limitations

This study has both strengths and limitations. Firstly, it demonstrates innovation in its objective to design data visualisation components of an interactive dashboard prototype for real-time suicide surveillance and detection of emerging suicide clusters. While dashboards have been applied extensively to public health surveillance, this approach has not yet been explored in real-time suicide surveillance, particularly incorporating aberration detection and mapping components.

Although the application of the prospective space-time scan statistic did not detect statistically significant suicide clusters in this study, this finding was predicted early in the present research due to the lack of current suicide mortality data available, while prospective analysis is limited to the identification of clusters that are currently occurring. This issue is one of the fundamental reasons for the establishment of the SSHO, to provide real-time suspected suicide data to detect emerging clusters and conduct predictive modelling of cluster occurrence. In the instance of national implementation of real-time suicide surveillance across Ireland, the level of data available for analysis would increase significantly from that analysed within the current study. Furthermore, the data would be captured in a timely manner ensuring up-to-date figures, thereby providing the optimal environment for the application of the prospective spatio-temporal scan statistic mode involving repeated daily analysis. In addition, there is a possibility that clusters that occurred over a prolonged period outside of the parameters applied within the present research were not detected. Furthermore, since SaTScan scan statistic is restricted to detecting circle-like clusters, clusters of irregular shapes may not have been detected. To overcome this obstacle, the possibility to employ a hybrid cluster detection method that integrates scanning techniques to detect both circular and irregularly shaped clusters in real-time could be explored in future research. Since no statistically significant cluster was detected by the scan statistic in the current study, the sensitivity and specificity of the statistical algorithm underpinning the dashboard must be further investigated to determine the possibility for false positive results that may trigger the initiation of a crisis response and subsequent deployment of resources unnecessarily. In terms of data visualisation features of the dashboard prototype, preliminary visualisation techniques were employed to determine the proof of concept. While the figures presented in the current study adequately present the analytical output, more sophisticated possibilities could be explored with the support of a software engineer to further enhance the quality of the dashboard display. Lastly, possible changes in the spatial distribution of the population at small area level during the study period were not controlled for.

### Implications for suicide prevention

The findings of this study have strong implications for suicide prevention. Firstly, the development and incorporation of an algorithmic intuitive interface for real-time suicide surveillance provides an early-warning system to facilitate implementation or activation of crisis plans to respond to emerging suicide clusters. It further provides an evidence-base to increase the capacity for early intervention when emerging clusters are detected, while also assisting with optimising resource allocation and informing health service responses in geographical areas with recurring clusters by means of the mapping feature.

An encrypted online platform housing real-time suspected suicide mortality data seeks to promote close collaboration between key stakeholders, not only for crisis response purposes, but to support longer-term suicide prevention initiatives such as means restriction efforts on new methods of concern or at identified locations where individuals frequently take their lives, as well as targeted interventions for identified vulnerable subpopulations *via* longitudinal trend analysis. Based on the population density of an area where an increase in cases of suspected suicide is observed, a threshold relative to that population size must be met to statistically detect a cluster. The period immediately following the detection of an emerging cluster would involve close monitoring of the population at-risk, as well as awareness-raising among services such as primary care and mental health services in preparation for possible activation of a crisis response plan including all key stakeholders.

In terms of policy implications, the development of a real-time suicide surveillance dashboard holds the potential to facilitate monitoring and evaluation of both suicide prevention strategies and wider mental health policies by providing the data infrastructure to track progress towards the aims and objectives of implementation plans (11, 37).

## Conclusion

Real-time suicide surveillance is a novel development in public health research and one that continues to evolve. Integration of a cluster detection approach involving space-time scan statistics, data visualisation techniques, and predictive modelling in a real-time suicide surveillance system would facilitate prospective detection of emerging clusters, at-risk populations, methods, and locations of concern. However, the validation of the system relies on current suicide mortality data availability.

Collectively, the components of the dashboard prototype demonstrate promising real-world applicability as a proactive monitoring tool for timely action in suicide prevention by facilitating informed planning and preparedness to respond to detected emerging suicide clusters, risk factors and other concerning trends identified by the SSHO.

## Data Availability

The datasets included in this study include individual level suicide mortality data to which data laws are not applicable. However, since the data used in this study cannot be shared publicly due to the inclusion of information that may be personally identifiable and may cause upset to the relatives of the deceased individuals. Requests to access the datasets should be directed to ruth.benson@ucc.ie.
